# Age-related patterns of vigorous-intensity physical activity in youth: The International Children's Accelerometry Database

**DOI:** 10.1016/j.pmedr.2016.05.006

**Published:** 2016-05-16

**Authors:** Kirsten Corder, Stephen J. Sharp, Andrew J. Atkin, Lars B. Andersen, Greet Cardon, Angie Page, Rachel Davey, Anders Grøntved, Pedro C. Hallal, Kathleen F. Janz, Katarzyna Kordas, Susi Kriemler, Jardena J. Puder, Luis B. Sardinha, Ulf Ekelund, Esther M.F. van Sluijs, on behalf of the International Children's Accelerometry Database (ICAD) Collaborators

**Affiliations:** a MRC Epidemiology Unit and UKCRC Centre for Diet and Activity Research (CEDAR), University of Cambridge School of Clinical Medicine, Box 285, Institute of Metabolic Science, Cambridge Biomedical Campus, Cambridge CB2 0QQ, United Kingdom; b Department of Sport Science and Clinical Biomechanics, University of Southern Denmark, Odense, Denmark; c Norwegian School of Sport Science, Oslo, Norway; d Department of Movement and Sports Sciences, Ghent University, Ghent, Belgium; e Centre for Exercise, Nutrition and Health Sciences, University of Bristol, Bristol, UK; f Centre for Research and Action in Public Health, University of Canberra, Canberra, Australia; g Federal University of Pelotas, Pelotas, Brazil; h Department of Health and Human Physiology, University of Iowa, Iowa City, US; i School of Social and Community Medicine, University of Bristol, UK; j Epidemiology, Biostatistics and Public Health Institute, University of Zürich, Switzerland; k Service of Endocrinology, Diabetes and Metabolism, Centre Hospitalier Universitaire Vaudois, University of Lausanne, Switzerland; l Division of Pediatric Endocrinology, Diabetology and Obesity, Centre Hospitalier Universitaire Vaudois (CHUV), Lausanne, Switzerland; m Exercise and Health Laboratory, Faculty of Human Movement, Technical University of Lisbon, Lisbon, Portugal

**Keywords:** ICAD, Motor activity, Child, Adolescent, Epidemiology

## Abstract

Physical activity declines during youth but most evidence reports on combined moderate and vigorous-intensity physical activity. We investigated how vigorous-intensity activity varies with age.

Cross-sectional data from 24,025 participants (5.0–18.0 y; from 20 studies in 10 countries obtained 2008–2010) providing ≥ 1 day accelerometer data (International Children's Accelerometry Database (ICAD)). Linear regression was used to investigate age-related patterns in vigorous-intensity activity; models included age (exposure), adjustments for monitor wear-time and study. Moderate-intensity activity was examined for comparison. Interactions were used to investigate whether the age/vigorous-activity association differed by sex, weight status, ethnicity, maternal education and region.

A 6.9% (95% CI 6.2, 7.5) relative reduction in mean vigorous-intensity activity with every year of age was observed; for moderate activity the relative reduction was 6.0% (5.6%, 6.4%). The age-related decrease in vigorous-intensity activity remained after adjustment for moderate activity. A larger age-related decrease in vigorous activity was observed for girls (− 10.7%) versus boys (− 2.9%), non-white (− 12.9% to − 9.4%) versus white individuals (− 6.1%), lowest maternal education (high school (− 2.0%)) versus college/university (ns) and for overweight/obese (− 6.1%) versus healthy-weight participants (− 8.1%). In addition to larger annual decreases in vigorous-intensity activity, overweight/obese individuals, girls and North Americans had comparatively lower average vigorous-intensity activity at 5.0–5.9 y.

Age-related declines in vigorous-intensity activity during youth appear relatively greater than those of moderate activity. However, due to a higher baseline, absolute moderate-intensity activity decreases more than vigorous. Overweight/obese individuals, girls, and North Americans appear especially in need of vigorous-intensity activity promotion due to low levels at 5.0–5.9 y and larger negative annual differences.

## 1 Background

Physical activity declines throughout childhood and adolescence (Dumith et al., 2011). Results from the International Children's Accelerometry Database, which includes data from studies in Europe, North America, Australia and Brazil, show a cross-sectional decrease of 4.2% in total physical activity with each additional year of age (Cooper et al., 2015). Longitudinal data indicates that over 10 min/day of physical activity every year is replaced by sedentary time between 9/10 y and 13/14 y among British children (Corder et al., 2013a). Investigations of physical activity for obesity prevention are somewhat inconclusive in young people (Steinbeck, 2008, Wareham et al., 2005) whereas evidence supporting physical activity for the reduction of cardiovascular disease risk in youth is more hopeful (Ekelund et al., 2012a, Janssen and Leblanc, 2010). Some evidence indicates that a 10-minute increase in moderate to vigorous-intensity physical activity (MVPA) is associated favorably with metabolic outcomes including a smaller waist circumference and lower fasting insulin among young people (Ekelund et al., 2012b).

The majority of available evidence describing physical activity change during childhood and adolescence focuses on combined MVPA, which may mask intensity-specific changes in activity over time (Corder et al., 2013b). It has been suggested that vigorous-intensity physical activity may be more important than lower intensity activity for weight control (Janssen and Ross, 2012, Steele et al., 2009) and has more predictive value for obesity among boys (Latt et al., 2015). Furthermore, a higher baseline BMI z-score has been associated with a greater decline in vigorous activity during weekends between the ages of 10 y and 11 y (Corder et al., 2013b). Vigorous-intensity physical activity accounts for the lowest proportion of overall physical activity among young people, and may decline more rapidly than any other activity intensity during adolescence (Corder et al., 2013b). Maintaining or increasing vigorous-intensity activity may require different health promotion strategies than interventions targeting lower intensity activity, such as promotion of organized sport versus lifestyle activities. Evidence regarding the pattern of vigorous-intensity physical activity throughout childhood and adolescence is inconclusive and more evidence is needed to reflect a wide age range and large population samples.

We investigated how vigorous-intensity physical activity varies with age among young people aged 5–18 years; we additionally compared this with the decline in moderate-intensity physical activity over the same period. We hypothesized that the decline in vigorous-intensity physical activity would be greater than that in moderate-intensity physical activity. We additionally investigated whether age-related differences in vigorous-intensity physical activity varied by sex, weight status, maternal education, ethnicity and geographic region to better understand how to target physical activity promotion among population subgroups.

## 2 Methods

### 2.1 Study

The International Children's Accelerometry Database (ICAD, http://www.mrc-epid.cam.ac.uk/Research/Studies/) was established to pool data on objectively measured physical activity from studies in youth worldwide. The aims, design, study selection, inclusion criteria, and methods of the ICAD project have been described in detail previously (Sherar et al., 2011). Briefly, in 2008 a PubMed search for potential contributors was undertaken. From this search, 19 studies using the same type of accelerometer (Actigraph) and including at least 400 participants aged 3 to 18 years were identified. Additional studies were identified by personal communication. In total, 25 studies were identified and approached, of which 21 contributed data to ICAD (Boyd et al., 2013, Sherar et al., 2011).

### 2.2 Participants

Previous analyses in ICAD indicated a linear association of MVPA with age from 5 to 18 years of age (Cooper et al., 2015); therefore only participants aged ≥ 5.0 years were included in these analyses. Due to the age criteria, the present analysis included data on children and adolescents (aged 5–18 years) from 20 of the 21 studies included in ICAD from Australia, Brazil, Europe, and North America, in which data on objectively measured physical activity were available (N = 24,025 participants). These studies were performed between 1998 and 2009. All contributing studies obtained relevant ethical approval.

### 2.3 Assessment of Physical Activity

A detailed description of the assessment of physical activity is available elsewhere (Sherar et al., 2011). Briefly, all physical activity measurements were made with uniaxial, waist-worn Actigraph accelerometers (models 7164, 71256 and GT1M); these accelerometers have been validated for assessment of free living physical activity energy expenditure in young people (Ekelund et al., 2001). All available raw accelerometer data from contributing studies were reanalysed to provide physical activity outcome variables that could be directly compared (KineSoft, version 3.3.20). For consistency across studies, data files were reintegrated to a 60-second epoch where necessary and non-wear time was defined as 60 min of consecutive zeros, allowing for 2 min of nonzero interruptions (Troiano et al., 2008). All children with at least 1 day of at least 500 min of measured monitor wear time between 7 am and midnight were included in this analysis. Evenson cut-points (Evenson et al., 2008) were used to estimate moderate- (≥ 2296 cpm) and vigorous-intensity (≥ 4012 cpm) physical activity. For descriptive purposes, time spent sedentary was defined as all minutes registering less than 100 cpm (Treuth et al., 2004).

### 2.4 Non-accelerometer variables

Height and weight were measured using standardized clinical procedures across studies. Body mass index (BMI, in kg/m^2^) was calculated and participants were categorized into normal weight or overweight/obese groups according to age and sex-specific cut points (Cole et al., 2000). As a proxy for socio-economic status, maternal education was used in three categories of up to completing high school, college or vocational training and university or higher. Participants were categorized into four groups to represent ethnicity: white, African ancestry, Hispanic and other (including Asian). Studies were grouped into five geographical areas (regions): the UK, the rest of Europe, North America, Australia and Brazil. Not all variables were available in all datasets; the studies in which they are available are shown in Table 1.

## 3 Statistics

Analyses were performed using STATA version 14.0 (Statacorp, College Station, TX).

The outcome variables were minutes per day of vigorous- or moderate-intensity physical activity which, due to non-normality, were (natural) log-transformed prior to linear regression analyses. Vigorous-intensity physical activity data over the 99th percentile were set to the 99th percentile value to remove extreme observations. Absolute vigorous- and moderate-intensity physical activity is also presented descriptively. Where multiple data were available from a participant, only the first measurement was included in the analyses.

Linear regression models were used to investigate age-related patterns in vigorous- and moderate-intensity physical activity. Models included age as the exposure, and were adjusted for monitor wear time and study. The beta coefficient for age was back transformed to represent a ratio of the geometric mean of vigorous-intensity physical activity for every year increase in age. Vigorous- and moderate-intensity physical activity were each modeled, with vigorous activity additionally adjusted for moderate-intensity activity. This adjusted model aimed to assess whether any age-related difference in vigorous-intensity physical activity was independent of moderate-intensity physical activity and therefore whether the components of MVPA differ with age independently.

For vigorous-intensity physical activity, interaction parameters were included to investigate potential differences in the effect of age by sex, weight status, ethnicity, maternal education (as a proxy for SES) and geographical region. If a significant interaction was found, models were stratified by subgroup to aid interpretation (Table 3). Interactions were not investigated for moderate activity as vigorous-intensity physical activity is the main focus of this analysis due to the emerging evidence on the importance of vigorous activity in respect to health, including weight control (Janssen and Ross, 2012, Steele et al., 2009).

To check our linearity assumption, analyses were repeated with age categorized into 2-year age bands (data not shown) and as the changes in log (vigorous-intensity physical activity) between adjacent age bands were similar, it is reasonable to assume that the age/physical activity association is log-linear across the 5–18 year age range.

Subsequently, to aid interpretation of the results, the mean(SD) of vigorous- and moderate-intensity physical activity among participants 5.0–5.9 years-old (n = 1303) are presented (Table 2, Table 3) and have been used to estimate the absolute age-related differences over 5–18 years (13 year period).

## 4 Results

Participant characteristics are summarized in Table 1. Every year increase in age was associated with a relative reduction in mean vigorous-intensity physical activity of 6.9% (95% CI 6.2%, 7.5%) and in mean moderate-intensity physical activity of 6.0% (95% CI 5.6%, 6.4%) (Table 2). The age-related difference in vigorous-intensity physical activity was substantially attenuated but remained significant when adjusted for moderate-intensity physical activity. Based on mean values for the 5.0–5.9 year-olds within ICAD, we estimated that vigorous-intensity physical activity would be 5.1 min at 18 years-old, representing a decrease of 7.9 min over 13 years.

There was evidence that the age/vigorous-intensity physical activity association significantly differed by sex, ethnicity, maternal education and weight status. A larger age-related decrease in vigorous-intensity physical activity was observed among groups with lower levels at baseline, in girls versus boys, and among overweight/obese versus healthy weight individuals. However, compared to white individuals, non-white individuals had higher vigorous-intensity physical activity at baseline and a larger age-related decrease; this was additionally seen for participants with low maternal education (high-school only) versus those whose parents had a college or university education. In UK studies, vigorous-intensity physical activity increased with age whereas a decrease with age was observed in studies from North America and Australia and no significant change among the rest of Europe.

## 5 Discussion

We estimated a 6.9% reduction in objectively-measured vigorous-intensity physical activity, relative to age 5 y, with every year of age during childhood and adolescence; representing a reduction of 7.8 min between the ages of 5 and 18. The relative decrease in moderate-intensity physical activity was slightly smaller at 6.0% per year of age, equivalent to a 22.8-minute reduction over the same time period. However, due to larger baseline values, in absolute terms the decline in moderate-intensity physical activity is greater than that of a vigorous-intensity. The age-related difference in vigorous-intensity physical activity was significant independent of moderate-intensity physical activity; indicating that within the previously cited decline of MVPA throughout childhood and adolescence (Cooper et al., 2015), the vigorous-intensity physical activity decline may be relatively greater.

There is relatively little data exclusively examining change in vigorous-intensity physical activity with which to compare our results. However, a 7% decrease in total physical activity per year during adolescence was concluded by a review including 26 longitudinal studies (Dumith et al., 2011). This review only found three studies examining change in vigorous-intensity physical activity and summarized the mean annual change across these three studies as − 5.2% (95% CI − 37.2 to 25.5) p = 0.51 (Dumith et al., 2011). As the authors state, there were likely to be insufficient data to adequately estimate change over time (Dumith et al., 2011). Nevertheless, that review highlighted the limited evidence available regarding the age-related change in vigorous-intensity physical activity and supports the need to examine this component separately from MVPA. Investigation of patterns of vigorous-intensity physical activity may be especially timely considering the increasing evidence suggesting associations of vigorous-intensity activity with anthropometric and cardiometabolic indicators (Gutin and Owens, 2011, Yin et al., 2012).

The age-related decreases in vigorous-intensity physical activity throughout adolescence appear greater for certain population subgroups, including girls, non-white participants, participants with low maternal education, overweight/obese individuals and participants from studies in North America and Australia. Regression to the mean appears to explain some of these differences, with population groups with higher vigorous-intensity physical activity at 5.0–5.9 years demonstrating larger negative differences; this appears to be the case for non-white individuals, those with lower maternal education and Australian children. Maternal education was used as a proxy for socio-economic status but the socio-economic patterning of activity is complex and may not be comparable across countries, especially those at different stages of their economic transition (Hallal et al., 2012). Other indicators of socio-economic status may show different associations with physical activity and further work examining patterns of activity change by different measures of socio-economic status would be useful.

Our results align with previous evidence indicating that girls are less active than boys, that overweight/obese individuals are less active than their normal weight peers (Cooper et al., 2015, Dumith et al., 2011, Hallal et al., 2012) and that North American youth may be particularly inactive (Cooper et al., 2015, Dumith et al., 2011, Hallal et al., 2012). However, alongside low initial vigorous-intensity physical activity levels among girls, overweight/obese individuals and participants in North American studies, our results show that these groups also appear to have larger negative annual differences in vigorous-intensity physical activity. Given the suggested greater health benefits of vigorous- over moderate-intensity physical activity (Janssen and Ross, 2012, Steele et al., 2009), these groups might therefore be especially in need of interventions to promote vigorous-intensity physical activity.

Results from UK studies suggest an age-related increase in vigorous-intensity physical activity, contrary to expectations and the results from other regions. To further investigate this, post-hoc analyses were conducted with each UK study separately and the positive nature of the age-related vigorous-intensity physical activity increase appears to be driven by one individual study (Gidlow et al., 2008); removal of this study changes the direction of the association (data not shown) and makes the results for UK studies consistent with those from North America and Australia. Possible explanations for the differences between this study and other British studies may include sampling strategy or seasonal timing (Atkin et al., 2015) of the measurements for this sample aged 3 to 16 years-old (Gidlow et al., 2008), which we were unable to account for in these analyses.

These results should be considered in the context of the whole physical activity intensity spectrum. Results indicated that age-related differences in vigorous-intensity physical activity were independent of moderate activity and therefore that vigorous-intensity physical activity may decrease disproportionately more with age within MVPA. It should be noted that our analyses did not take differing levels of light-intensity physical activity or sedentary time into account. When we examine the descriptive data, and also consider other recent publications (Cooper et al., 2015), on a population level, most of the volume of the physical activity decline appears to consist of light-intensity activity being replaced by sedentary time. Nevertheless, it is likely that physical activity promotion should continue to include focus on the promotion and maintenance of vigorous-intensity physical activity. The physical activity promotion strategies needed to promote activity at each end of the intensity spectrum are likely to vary, as is the approach needed for different population subgroups. However, our results suggest that promotion of vigorous-intensity physical activity may warrant further attention and especially among certain population subgroups such as girls and overweight/obese individuals.

We extrapolated our results to estimate the potential magnitude of the age-related difference in vigorous physical activity throughout adolescence, however, these results are cross-sectional so should be interpreted as such. There are some limitations with the ICAD data in that it may not be representative of national populations and that the data over-represents certain countries such as the UK and the US. Additionally, certain age-groups are under-represented with adolescents ≥ 14 years-old representing < 9% of the total number of participants in this analysis; this is reflective of the limited physical activity data available for older adolescents (Corder et al., 2015). We only included the first data point from each individual; we deemed it necessary to remove follow-up intervention data from this analysis but we acknowledge that baseline data from longitudinal studies may have been collected earlier than some cross-sectional studies which may contribute to potential temporal bias. The data sources within ICAD have been collected over a wide time-interval (about 20 years) and estimated age-effects may therefore have been influenced by possible temporal trends in activity over the time-period of data-collection. As age-intervals in each data source are non-randomly distributed over this time-interval, this may introduce bias which the adjustment for study in our analysis is unlikely to prevent. Potential future additions to the ICAD dataset should allow a more comprehensive analysis of intra-individual physical activity change. We did not include data on maturation in our analysis; this was primarily due to the relatively small number of studies having comparable data on maturation. Nonetheless, this analysis uses data from a very large sample of young people over a wide age range from four continents so we can be relatively confident in our results. Waist worn accelerometers may have limitations when assessing certain types of physical activity such as cycling and must be removed for swimming and other aquatic activities (Corder et al., 2007a); this may lead to bias if engagement of these activities differs across the age range or between population groups. Data within ICAD is available in 60-second epochs and compared to shorter epoch lengths, this may reduce the amount of vigorous-intensity physical activity recorded and therefore influence the variability of the outcome (Corder et al., 2007b). As physical activity is more erratic and vigorous-intensity physical activity more short-lasting at a young age (Oliver et al., 2007), this longer epoch may result in underestimation of vigorous-intensity activity at age 5 and of the annual decline. For example, free-living Actigraph assessed vigorous-intensity activity (mean min/day) in a group of 27 adolescents 15.8(0.6) years-old was 0.4(1.1)min/day when the data was aggregated to a 60 second epoch versus 2.3(2.5)min/day when using the same data with the original 5-second epoch (Corder et al., 2007b).

Vigorous-intensity physical activity appears to fall by 6.9% with each year of age during childhood and adolescence. Within MVPA, vigorous-intensity physical activity appears to decline proportionately more than moderate-intensity physical activity. However, in absolute terms, the moderate-intensity physical activity decline is greater than that of vigorous-intensity physical activity. The age-related differences in vigorous-intensity physical activity appear to be greatest among girls, non-white individuals, overweight and obese participants and those from North America and Australia. Due to low levels of vigorous-intensity physical activity in young childhood and large age-related differences, girls, overweight and obese individuals and young people from North America appear most in need of vigorous-intensity physical activity promotion.

## Conflict of interest

Kirsten Corder reports receiving the following grants: Lead Applicant - A cluster randomised controlled trial to evaluate the effectiveness and cost-effectiveness of the GoActive program to increase physical activity among 13–14 year-old adolescents. Project: 13/90/18 National Institute for Health Research Public Health Research Program Sept. 2015 – Feb. 2019. Co-Applicant - Opportunities within the school environment to shift the distribution of activity intensity in adolescents. Department of Health Policy Research Program. Dec. 2013–Nov. 2016. Kirsten Corder is a Director of Ridgepoint Consulting Limited, an operational improvement consultancy.

Data from studies in Europe, North America, Australia and Brazil and obtained 2008–2010.

## Figures and Tables

**Table 1:** Descriptive characteristics of included participants from 20 studies.

	Studies	N	Descriptive data
Sex	1–20	24,025	
N (%) Boys			10,756 (44.7)
N (%) Girls			13,279 (55.3)
Age (years)	1–20	24,025	
Mean (SD)			11.02 (2.63)
Range			5.0–18.0
Location	1–20	24,025	
N (%) UK			9767 (40.7)
N (%) Continental Europe			4350 (18.1)
N (%) US			6973 (29.0)
N (%) Brazil			455 (1.9)
N (%) Australia			2480 (10.3)
Weight status	1–20	19,705	
N (%) Overweight/obese			2002 (10.2)
Ethnicity	5, 8, 10–13, 15, 17, 18	10,219	
N (%) White			5344 (52.3)
N (%) Black			2081 (20.4)
N (%) Hispanic			2109 (20.6)
N (%) Other			685 (6.70)
Maternal education	1–3, 5–8, 10, 12, 14, 16, 19	11,916	
N (%) ≥ High school			6271 (52.6)
N (%) College			1975 (16.6)
N (%) ≥ University			3670 (30.8)
Physical activity (min/day)	1–20	24,025	
VPA (Median [IQR])			10.6 [5.2, 19.5]
MPA (Median [IQR])			34.25 [23.0, 47.9]
Wear time (Mean(SD))			769.7 (88.6)
Sedentary (Mean(SD))			349.1 (103.2)

VPA Vigorous-intensity physical activity.

MPA Moderate-intensity physical activity.

Maternal education: Mothers highest education 1 “up to and including high school” 2 “college vocational training” 3 “University +”.

Studies: 1 “ALSPAC” (Boyd et al., 2013) the study includes a searchable data dictionary 2 “Belgium Pre-School Study” (Cardon and De Bourdeaudhuij, 2007) 3 “CLAN” (Crawford et al., 2010) 4 “CSCIS” (Eiberg et al., 2005) 5 “Denmark EYHS” (Brage et al., 2004) 6 “Estonia EYHS” (Riddoch et al., 2005) 7 “HEAPS” (Salmon et al., 2006) 8 “IOWA” (Janz et al., 2001) 9 “NHANES 2005–6” (Centers for Disease Control and Prevention, 2005) 10 “Norway EYHS” (Ommundsen et al., 2006) 11 “NHANES 2003–4” (Centers for Disease Control and Prevention, 2005) 12 “PEACH” (Page et al., 2009) 13 “Pelotas” (Victora et al., 2008) 14 “Portugal EYHS” (Sardinha et al., 2008) 15 “SPEEDY” (van Sluijs et al., 2008) 16 “Project TAAG” (Stevens et al., 2005) 17 “CHAMPS UK” (Gidlow et al., 2008) 18 “Ballabeina Study” (Niederer et al., 2009) 19 “KISS” (Zahner et al., 2006) 20 “CHAMPS US” (Pfeiffer et al., 2009).

**Table 2:** Estimated associations of age (years) with VPA and MPA from linear regression models.

	Ratio	95% CI	P value	% relative decrease/increase in geometric mean (95% CI)	Mean (SD) at 5.0–5.9 y	Extrapolated change 5-18 y (min)
Single models						
VPA	0.931	(0.925, 0.938)	< 0.001	− 6.9% (6.2%, 7.5%)	12.97 (12.64)	− 7.85
MPA	0.940	(0.936, 0.944)	< 0.001	− 6.0% (5.6%, 6.4%)	41.32 (17.46)	− 22.83
Adjusted						
VPA	0.989	(0.984, 0.994)	< 0.001	− 1.1% (0.6%, 1.6%)		
MPA	1.036	(1.035, 1.036)	< 0.001	+ 3.6% (3.5%, 3.6%)		

Adjusted for monitor wear time with study as a covariate.

95% CI; 95% confidence interval.

% diff; calculated from the ratio as the % relative decrease/increase in geometric mean of VPA with age.

Ratio; ratio of the geometric mean VPA for every year increase in age.

VPA; Vigorous-intensity physical activity (min).

MPA; moderate-intensity physical activity (min).

Data from 20 studies in Europe, North America, Australia and Brazil and obtained 2008–2010.

**Table 3:** Estimated associations of age (years) with VPA from linear regression models. Results are presented within subgroups for which the p-values for interactions with age were p < 0.05.

Subgroups	Interaction (p value)	Ratio	95% CI	P	% relative decrease/increase in geometric mean (95% CI)	Mean (SD) VPA min at 5.0–5.9 y	Extrapolated change 5-18 y (min)
**Sex**	< 0.001						
Male		0.971	(0.962, 0.980)	< 0.001	− 2.9% (2.0%, 3.8%)	15.16 (15.03)	− 4.82
Female		0.893	(0.884, 0.901)	< 0.001	− 10.7% (9.9%, 11.6%)	10.98 (9.56)	− 8.46
**Ethnicity**	< 0.001						
White		0.939	(0.926, 0.954)	< 0.001	− 6.1% (6.6%, 7.4%)	10.58 (8.43)	
Black		0.871	(0.856, 0.886)	< 0.001	− 12.9% (11.4%, 16.4%)	*	
Hispanic		0.906	(0.982, 0.921)	< 0.001	− 9.4% (7.9%, 1.8%)	*	
Other		0.906	(0.871, 0.942)	< 0.001	− 9.4% (12.9%, 5.8%)	*	
**Maternal education**	0.005						
High school		0.980	(0.964, 0.996)	0.013	− 2.0% (0.04%, 3.6%)	17.91 (14.9)	− 4.14
College		0.979	(0.953, 1.005)	0.112			
University		1.004	(0.984, 1.025)	0.667			
**Weight status**	0.001						
Normal		0.939	(0.931, 0.946)	< 0.001	− 6.1% (5.4%, 6.9%)	12.85 (12.34)	− 7.18
o/o		0.919	(0.900, 0.938)	< 0.001	− 8.1% (6.2%, 10.0%)	11.71 (10.16)	− 7.80
**Region**							
UK	ref	1.065	(1.041, 1.089)	< 0.001	+ 6.5% (4.1%, 8.9%)	13.60 (12.45)	+ 17.24
Europe	0.001	1.016	(0.997, 1.035)	0.095			
North America	< 0.001	0.885	(0.877, 0.894)	< 0.001	− 11.5% (10.6%, 12.3%)	11.18 (8.31)	− 8.90
Brazil	0.004	0.673	(0.448, 1.011)	0.056			
Australia	< 0.001	0.979	(0.867, 0.992)	0.001	− 2.1% (0.8%, 13.3%)	19.78 (16.06)	− 4.78

Adjusted for monitor wear time and study.

95% CI; 95% confidence interval.

% diff; calculated from the ratio as the % relative decrease/increase in geometric mean of VPA with age.

Ratio; ratio of the geometric mean VPA for every year increase in age.

VPA; Vigorous-intensity physical activity (min).

o/o; overweight or obese individuals.

* Insufficient data to provide meaningful mean value (N < 10).

Extrapolated change calculated as [(Ratio^13)*Mean at 5–5.9 years-old]-Mean at 5–5.9 years-old.

Data from 20 studies in Europe, North America, Australia and Brazil and obtained 2008–2010.
